# Parenteral Antimicrobial Treatment Diminishes Fecal *Bifidobacterium* Quantity but Has No Impact on Health in Neonatal Dairy Calves: Data From a Field Trial

**DOI:** 10.3389/fvets.2021.637271

**Published:** 2021-03-31

**Authors:** Olivia C. O'Keefe, Dale A. Moore, Craig S. McConnel, William M. Sischo

**Affiliations:** Field Disease Investigation Unit, Department of Veterinary Clinical Sciences, Washington State University, Pullman, WA, United States

**Keywords:** *Bifidobacterium*, dairy, calf, antimicrobial, ampicillin, diarrhea

## Abstract

There is evidence that neonatal calves are over treated with antimicrobials that may disrupt colonization of their gastrointestinal tract (GIT) microbiota. The study objectives were to assess the decision-making process of antimicrobial use on a commercial dairy and impacts of parenteral antibiotics on dairy calves' GIT *Bifidobacterium* and calf health. Unhealthy pre-weaned dairy calves were enrolled based on farm personnel identification with age-matched healthy calves. Half the calves in each group were treated with a 3-day course of IM ampicillin and half were given supportive therapy as needed. Health scores (appetite, fecal consistency, attitude, and temperature) were recorded twice daily throughout the study. Because of inconsistency in employee health decisions, the 121 enrolled calves were reassessed using objective clinical observations plus fecal dry matter and placed into 1 of 3 health categories: healthy, uncomplicated diarrhea (bright attitude and good appetite but with diarrhea), and sick. Accounting for treatment group allocation, this resulted in six post-enrollment health and treatment categories. Calves were followed daily for 14 days post-enrollment and fecal samples collected at 6 time points and *Bifidobacterium* was quantified from these samples using quantitative PCR. The objective criteria for disease definition reclassified many “unhealthy” calves as uncomplicated diarrhea. Including all calves, on average, the quantity of *Bifidobacterium* decreased from the day of enrollment (median 8 days of age) across time to 14 days post-enrollment. Calves given an antibiotic the day of enrollment had a greater decrease in *Bifidobacterium* 4 and 9 days later relative to enrollment *Bifidobacterium* compared to untreated calves. At enrollment, sick calves and those categorized as uncomplicated diarrhea were more likely to have low *Bifidobacterium* counts and less likely to be categorized as healthy following antimicrobial treatment. Our results indicate that relying on farm personnel to identify morbidity may lead to some clinical misclassification. There was no indication that antimicrobials affected subsequent health outcomes, but antimicrobials did impact *Bifidobacterium* dynamics. These results highlight the importance and difficulty in assigning appropriate illness classification on farms and point to a need to develop better point of care diagnostics that improve calf husbandry and stewardship of antimicrobials.

## Introduction

Antimicrobials are a common tool used to manage calf health and treat pre-weaning calf diseases. From a U.S, national survey of heifer rearing nearly all calves with respiratory signs and 75% of calves observed with diarrhea were treated with an antimicrobial (1). In a study involving calf raising facilities, 82% of calves observed with respiratory signs and 73% of calves observed with diarrhea were treated with an antimicrobial (2). This has led to discussions about the appropriate use of antimicrobials to treat disease in pre-weaned calves and whether diarrhea (a common reason that an antimicrobial is administered to a calf) is a symptom in a disease spectrum rather than a disease (3). One part of the discussion is that severe diarrhea should receive an antimicrobial to prevent septicemia and reduce mortality (4). A second part of the discussion is that GI disease identified only by observed diarrhea is over diagnosed and consequently antimicrobials are overused. In these cases, supportive therapy should be the first line treatment rather than antimicrobials (5, 6). While investigators have collected data regarding the frequency and type of antibiotics used to treat calves, little is known about the on-farm decision making process regarding calf health, the decision-making process for using antimicrobials, or the consequences of overuse. While there is evidence that overuse of antimicrobials is associated with diarrhea (6), little is known about overuse of antimicrobials on animal health over time, productivity, or gut microbiome subsequent or concomitant to treatment.

After birth, the neonatal GIT is rapidly colonized with a wide array of microorganisms and transitions as the animal matures. The transition to a stable GIT microbiota occurs early on in life; the exact age it occurs depends on the species. In dairy calves, there is an increase in the diversity and stability of the GIT microbiota over the first few months of age (7). This colonization is critical as interaction between the microbiota and the animal plays a key role in the development of the mucosal immune system (8) and is linked to resistance or susceptibility to diseases later in life. This suggests that the relationship between the GIT and immune system is most impacted in early life when the microbiome colonization of the GIT is variable (9). For cattle, if the normal developmental process of the intestinal microbiota in early life is disturbed, there may be long lasting health effects to the host (8) and downstream production performance (10).

There are data suggesting that antimicrobials impact the GIT and have negative health outcomes. Children treated with antimicrobials within the first 6 months of life are associated with an increased susceptibility to allergies, asthma, wheezing, eczema, inflammatory bowel disease, obesity, and type 2 diabetes mellitus later in life (9) It has been reported that intrapartum antibiotics resulted in altered microbiome in infants in the first weeks of life (11). When multiple antibiotics were given intrapartum, infants had lower GIT diversity as well as different bacterial communities at 6 weeks (12). There is evidence demonstrating the detrimental effects of antibiotic exposure in early life on the developing GIT as well as gastrointestinal microbiota composition in the adult (8, 11, 12). In calves, a study evaluating feeding low concentrations of antibiotics suggested they impacted relative abundance of genes coding for microbial cell functions and increased relative abundance of antibiotic resistance genes (13). Another study found no effect of oral antibiotics on ruminal microbiome (14). A study evaluating therapeutic and subtherapeutic oral oxytetracycline found a transient effect on the microbiome in the therapeutic group but observed no impact of subtherapeutic oxytetracycline on the microbiome. Differences between calves was mainly attributed to temporal changes across sampling times likely reflecting normal maturation (15).

*Bifidobacterium* species have been identified in the human health literature as critical members of the GIT microbiota with important functions within the colon that are associated with host health (16). Decreased abundance of these bacteria has been associated with diarrhea, obesity, and allergies. They also appear to support maturation of the immune system, support gut barrier functions, and protect against pathogens. Because it is presumed that *Bifidobacterium* plays a similar role in calves there are studies investigating probiotic feeding and its impact on GIT *Bifidobacterium* levels and health. Calves fed a supplemental bifidobacterial probiotic in an extensive housing system with their dams and that received mainly whole milk showed persistent, high levels of *Bifidobacterium* compared to calves also supplemented but reared in an intensive system without their dam and received a milk plus a supplemented concentrate diet (17). Although the study was confounded using different calf breeds in the two systems, the authors suggested that a pre-dominant milk diet influenced the persistence of fed supplemental bifidobacterial probiotics. As indirect evidence for probiotic impact on the microbiome, calves fed a multispecies probiotic of bacteria including *Bifdobacterium* spp. at the onset of diarrhea had faster resolution of diarrhea, but there was no difference in average daily weight gain (ADG) compared to placebo-treated control calves, suggesting only a short term effect of supplementation (18). Another study supporting the previous finding showed that probiotics had little to no impact on ADG or feeding behavior (19). Studies have also shown that probiotic supplementation resulted in a transient increase in abundance of *Bifidobacterium* spp. and was associated with fewer *E. coli* in calves' GIT and overall good health (20, 21). Another study showed that colostrum changed the GIT microbial community and enhanced the abundance of *Bifidobacterium* (22). While it appears that management to support and enhance *Bifidobacterium* exists there is a research gap in how other management interventions, particularly parenteral antimicrobials might impact GIT microbiota in calves and specifically *Bifidobacterium* spp.

The objectives of this study were to investigate the effect of parenteral treatment of healthy and unhealthy pre-weaned calves with antimicrobials on objective measures of pre-weaned calf health, growth, subsequent reproduction and the dynamics of fecal *Bifidobacterium* spp. The hypotheses were that parenteral antimicrobial treatment would negatively impact calf health, growth, and reproduction as well as dampen the normal dynamics of fecal *Bifidobacterium*, though these effects may be conditional on the health status of treated calves. In addition, we investigated the relationship between objective measures of pre-weaned calf health with decisions made by on-farm calf caretakers and their assessment of calf health.

## Materials and Methods

### Ethics Statement

The research protocol was reviewed and approved by the Institutional Animal Care and use Committee of Washington State University (ASAF 04925). All protocols involving calves housed on the commercial dairy farm were authorized by the farm owner, who was aware of all procedures.

### Study Design and Calf Enrollment

The study was conducted on a commercial dairy farm in the Pacific Northwest, USA. The farm milked 3,000 Holstein and Holstein-Jersey mix cows and raised all their replacement heifers. Study personnel worked with on-farm staff to identify animals for enrollment and conduct the study. All heifer calves born on the farm were fed previously collected and frozen, single source colostrum (3.8 L) within 2 h of calving and transferred within 24–48 h to the calf rearing facility that was separate but part of the dairy property. All calves entering the calf housing area were eligible to participate in the study unless they were involved in a dystocia, twin birth, or limb abnormality. Calf body weight was recorded at 24–48 h of age (median age = 24 h). At the same time, blood samples were obtained via jugular venipuncture to assess passive transfer of immunity by measuring total serum protein (TSP). From these blood samples, serum was obtained, and TSP values measured using a calibrated, clinical refractometer. Calves with TSP concentrations <5.2 g/dL indicated failure of transfer of passive immunity and were excluded from the study (23). For the study, TSP was summarized using quartiles and two categories created, low (as below the 25th percentile) and adequate (above the 25th percentile).

On-farm personnel were responsible for all the primary care of calves including feeding, cleaning, watering, bedding maintenance, and health assessments. This work involved three employees and the two employees tasked with the full-time care of calves had worked with calves on this farm for more than 5 years. One person was responsible for health decisions and feeding and cleaning protocols 6-days per week, one person supported feeding and cleaning 5-days per week and was responsible for health decisions 1-day per week, and one person supported feeding and cleaning 2-days per week (filling in for the regular team on their days off). Calves were housed in individual hutches with straw bedding that was renewed weekly and fed ~2.8 liters of whole unpasteurized milk from the farm's bulk tank milk dispensed into a bucket twice daily. Calves had ad-libitum access to grass hay and a grain-based starter feed mixture beginning at 4 days after birth. The starter feed was a farm-made ration that was 10% forage (generally grass hay) and 90% concentrate consisting of ground corn ears, corn dried distillers' grains, canola and soybean meal plus molasses to achieve a crude protein level of 25%. Water was available between milk feedings.

The study was designed to enroll eligible Holstein or Holstein-cross heifers at the first sign of disease (unhealthy). Simultaneously, an age-matched heifer with no clinical signs (healthy) was also enrolled. These initial health status decisions were made by farm personnel following the morning milk feeding. Although calf caretakers received *ad-hoc*, on the job training and a veterinarian was available to answer questions, the farm did not have specific protocols for assessing calf health and calves were identified as either unhealthy or healthy primarily based on workers' experience and supporting visual health observations such as attitude, appetite, posture, stool consistency, and risk age. Study personnel randomly allocated (using a pre-generated list) calves in each worker-identified group (healthy and unhealthy) to be treated by calf caretakers with either 3 mg/kg ampicillin trihydrate (Polyflex, Boehringer Ingelheim Vetmedica, Inc.) by intramuscular (IM) injection and 2.8 L oral bottle-fed electrolytes (Calva Lyte™, Calva Products LLC, Acampo CA) or given oral electrolytes alone. Based on employee discretion, calves could receive an ancillary therapy of bismuth subsalicylate. This resulted in an initial 4 study groups: (1) unhealthy-treated with an antimicrobial, (2) healthy-with no antimicrobial treatments, (3) healthy-treated with an antimicrobial, and (4) unhealthy-with no antimicrobial treatment. Based on farm protocols, calves enrolled in the antibiotic treatment groups were treated at enrollment and at 24-h intervals for a total of 3 treatment days. The choice of antimicrobial and protocol for administering it were a farm decision. If at any point in the study a calf demonstrated declining health indicated by an elevated body temperature (≥39.4°C) with decreased appetite and dull or depressed attitude, the calf was dropped from the study and was medically treated by farm personnel. On-farm personnel were not blinded to calf treatment group assignments.

### Data Collection

#### Fecal Samples

Because enrollment in the study (E1) did not begin until calves were identified as unhealthy and to ensure that we had a fecal sample from the day prior to enrollment (E0), commencing at 24–48 h post-parturition (P2) and daily thereafter, fecal samples from all calves eligible to be enrolled into the study were collected by digital rectal stimulation into sterile sampling bags (Thermo Fisher Scientific, USA). These samples were frozen on dry ice on the farm and subsequently transferred to the laboratory and stored at −80°C. As calves were enrolled into the trial as unhealthy or healthy controls, fecal samples were collected on enrollment day (E1), 4 days post-enrollment and the day following the final day of antimicrobial treatment (E4), 9 days post-enrollment (E9), and 14 days post-enrollment (E14). In addition to those samples, fecal samples analyzed in the study included the P2 and E0 samples.

#### Health Assessment

Twice daily, prior to feeding, study personnel blinded to calf group assignment independently observed all eligible and enrolled calves and recorded a series of assessments including: attitude (A = alert; AS = alert and sternal; D = dull/depressed; NA = non-responsive), a visual assessment of fecal consistency as observed from outside the hutch (0 = well-formed fecal samples; 1 = semi-formed fecal samples; 2 = loose fecal samples; 3 = watery fecal samples), and respiratory signs (normal, eye discharge, nasal discharge, and spontaneous cough). After the AM and PM feedings, appetite was scored (Y = good appetite, finished milk; N = did not finish milk; S = slow to finish milk; T = tube fed). Assessments began at P2 through E14. Rectal temperature was recorded for all calves at E1 and subsequently on sampling days E4, E9, and E14. Using on-farm records (Dairy Comp 305, VAS, Tulare CA), study calves were followed through to their first calving.

#### Fecal Dry Matter

Fecal samples collected at P2, E0, E1, E4, E9, and E14 were assessed for total dry matter by weighing out 2.5 grams of raw sample and drying the sample in an incubator at 25°C for 24 h. Percent dry matter was calculated as the difference between dry weight and wet weight divided by wet weight and multiplied by 100.

#### Average Daily Gain

All eligible calves were weighed (in pounds and converted to kg) at P2 using a balance calf scale (Paul Scale, Livestock Systems, Duncan OK, USA) that was calibrated with free weights before each use. At weaning (average = 57 days old), all enrolled calves were weighed again, and weaning age noted. Average daily gain was the difference between weaning weight and P2 weight relative to the weaning age (days). For analyses using P2 calf weight, it was summarized using quantiles and three categories created as below the 25th percentile, within the interquartile range (IQR), and above the 75th percentile.

#### Bacterial DNA Extraction From Fecal Samples and qPCR to Quantify Bifidobacterium

Bacterial DNA was extracted from fecal samples using the MagMAX^TM^ Total Nucleic Acid Isolation kit (Thermo Fisher Scientific, USA). Briefly, frozen fecal samples were thawed at room temperature, manually mixed and 300 mg of this sample was removed and suspended in 1 ml of PBS. This suspension was centrifuged at 100 RPM for 1 min to pellet gross solids. After centrifugation, 175 μl of the supernatant was removed and added to 235 μl lysis buffer provided by the kit manufacturer using a bead tube. This mixture was homogenized using a bead mill (Bead Mill, Fisher Scientific). The homogenized sample was centrifuged at 16,000 g for 10 min and 300 μl of the supernatant was removed and centrifuged at 16,000 g for 10 min to clarify. Following this centrifugation step, 115 μl was removed and transferred to the MagMax DNA extraction plate. Isolation was completed following manufacturer's directions in conjunction with the MagMax Express automated system (Applied BioSystems, USA). The final volume of extracted DNA was 90 μL.

The heat shock proteins in bacteria are highly conserved proteins and specific to bacterial genus and species, including the *gro*ES gene. Identification and quantification of *Bifidobacterium* spp. was carried out using qPCR targeting the bifidobacterial specific *gro*ES gene. The following oligonucleotide sequences were used to detect *gro*ES: gro-1 (5-CTCACACCGTTGGAAG-3) (forward) and gro-2 (5-GN(CA)GGAGACGATGAGGTA-3) (reverse) (24). A single qPCR reaction was performed containing 10 μL SsoAdvanced Universal SYBER Green Supermix (BioRad, USA), 1 μL forward and reverse primers (5 μM stock solution), 6 μL PCR nuclease-free water (Thermo Fisher Scientific, USA), and 2 μL fecal DNA template.

Quantification of PCR product was estimated from a standard curve developed from a sequence confirmed *Bifidobacterium longum* (Q349). Briefly, DNA was extracted from Q349 using a 5% chelex resin following a boil cell lysate procedure and the *gro*ES gene was amplified using PCR to obtain amplicons for cloning. PCR products were purified using QuiQuick PCR Purification Kit (Qiagen, MD). Cloning of our target sequence was done using a TOPO TA cloning kit dual promoter pCRII-TOPT vector (Invitrogen-ThermoFischer Scientific, MA, USA) per manufacturer's instructions using a One Shot™ TOP10 chemically competent *E*. *coli* (Invitrogen) as the host. Transformed cells were plated to LB agar containing 50 μg/ml of ampicillin (imMedia AMP Agar Invitrogen Q60120) and 40 mg/ml of X-Gal. Plates were incubated overnight at 37°C. Following incubation, 2–6 white or light blue colonies were selected and transferred to LB medium containing 50 μg/ml of ampicillin. Plasmid prep on culture was performed using Qiagen Plasmid Max prep kit (Qiagen 12163, Qiagen, USA). Transformation was confirmed by PCR. The number of copies were calculated using a portion of the transformed cells stock solution to create a standard curve (10^2^-10^8^, 7-points). Copies were calculated using the formula in the TOPO TA cloning kit protocol (Invitrogen, USA). Stock solution of transformed cells were stored in a 20% glycerol solution and kept frozen at −80C.

Amplification reactions were performed on an ABI StepOne Plus real time instrument (Applied Biosystems, USA). Amplification was carried out at 95°C for 1 min followed by 95°C for 30 s and 60°C for 30 s for a total of 40 cycles. Quantification estimates were generated based on the values generated from the standard curve and using the StepOne Plus 2.3v software (Applied BioSystems, USA). These estimates were adjusted to reflect copy number/gram of feces (copies/gm). Samples with a melt temperature between 87°C and 90.9°C but no amplification by 40 cycles were deemed to reflect a positive sample and used in analyses by randomly assigning a value between 0 and level of detection for the assay (10^2^ target copies). All samples, including external standards and non-template control, were run in duplicate.

### Data Analysis

#### Sample Size

Sample size was based on detecting differences in the temporal pattern of *Bifidobacterium* in the pre-weaning period. The assumptions for sample size determination were observing at least a 2 log_10_ difference in change of *Bifidobacterium* between time points and conditional on calf antimicrobial treatment status at E1 with an α and β error of 0.1. Based on experience, we assumed a variance of 3.6 log_10_. Sample size was calculated using R (R Project for Statistical Computing Version 4.0.2) package pwr. Estimated sample size was 56 calves per group and assuming a 10% loss to follow-up we determined a total sample of at least 61 calves per treatment group.

#### Disease Categories

The original enrollment criteria for the study as “unhealthy” and “healthy” were based on decisions made by farm personnel and these enrolled calves were then randomly assigned to an antibiotic treatment or supportive care only category. In parallel, all enrolled calves were independently assessed for appetite and pre-meal attitude by study personnel and a fecal sample collected and dry matter determined. Rectal temperature was measured on all calves identified as “unhealthy” by farm personnel. In addition, calves were evaluated for respiratory signs and few calves were identified with either ocular or nasal discharge and none were observed with otitis or voluntary cough. These data (excluding observations of respiratory signs) were used to create three post-enrollment health categories (Table 1). These categories were then used to classify calf health at all the sampling time points (P2, E0, E1, E4, E9, and E14) in all data analyses.

**Table 1:** Post-enrollment health score criteria for categorizing healthy and sick calves at enrollment during pre-weaned period.

**Health variable**	**Health category**		
	**Healthy**	**Uncomplicated diarrhea**	**Sick^a^**
Attitude^b^	Alert or alert-sternal	Alert or alert-sternal	Dull/depressed or non-responsive
Fecal DM^c^	DM >17.0%	DM ≤17.0%	DM ≤17.0%
Appetite^d^	Finished milk meal	Finished milk meal	Did not finish milk meal, or slow to finish milk meal, or milk meal fed via esophageal feeder
Rectal temperature	<39.4°C	<39.4°C	≥39.4°C

a *Calf was classified as sick if DM ≤17% and one other abnormal clinical sign or any single or combination of abnormal attitude, appetite, or rectal temperature*.

b *Observation of attitude prior to feeding*.

c *DM, Dry matter*.

d *Appetite at meal prior to enrollment*.

#### Summarizing and Modeling Changes in fecal Bifidobacterium Across Study Follow-Up

From qPCR findings, results were standardized to copy number per gram of fecal material (copies/gm) and log_10_ transformed. Means, minimum, maximum, medians, interquartile ranges, and contingency tables were determined to assess data distributions and make simple comparisons. Log_10_ *Bifidobacterium* (copies/gm) were summarized at each sampling time and compared using R and packages lme4 and emmean to calculate estimated marginal means. Temporal changes in log_10_ *Bifidobacterium* qPCR quantity between sampling times (E4, E9, and E14) and enrollment (E1) were determined and these differences summarized using quartiles and difference categories at each time point were developed based on below the 25th percentile, IQR, and above the 75th percentile and used as outcomes in multinomial logistic regression (R package, nnet). Multinomial logistic regression was used to assess relationships between temporal changes in log_10_ *Bifidobacterium* as the outcome. Initial models included risk factors associated with P2 (TSP categories, breed, and birth weight categories). Additional risk factors included in the initial models included those associated with E1 (antimicrobial exposure and health category), health categories on a sampling date as well as health category at prior sampling times, and interactions between E1 antimicrobial exposure and health categories. The goal for final models was to include risk factors or exposure variables associated with parsimonious models guided by AIC and improving residual deviance. Because antimicrobial exposure at E1 was the main effect evaluated in our study, it was retained in all models. Odds ratios (OR) with their 90% confidence intervals are reported.

#### Summarizing and Modeling Health Categories Across Study Follow-Up

Calves were assigned to health categories based on criteria shown in Table 1 independently for each sampling time point. These categories were used as outcomes in a set of multinomial logistic regression models (R project, nnet) that assessed risk factors for health for sampling points E1, E4, E9, and E14. Initial models included risk factors associated with P2 (TSP category, breed, and birth weight category), those associated with E1 (antimicrobial exposure and health category), and factors associated with the sampling date including log_10_ *Bifidobacterium* copies/gm categories, bismuth as an ancillary therapy, and health category at prior sampling times. Log_10_ *Bifidobacterium* was summarized for each sampling time using quartiles to create three categories (unique to each sampling time): below the 25th percentile, IQR, and above the 75th percentile. The goal for final models was to include risk factors or exposure variables associated with parsimonious models guided by AIC and improving residual deviance. Because antimicrobial exposure at E1 was the main effect evaluated in our study, it was retained in all models. OR and their 90% confidence intervals are reported.

#### Modeling Pre-weaning Average Daily Gain

Pre-weaning average daily gain (ADG) was calculated as the difference between weaning weight and P2 weight divided by weaning age (days). Because we were most interested in describing associations with low performers relative to high performers, ADG was summarized using quartiles and three categories created based on ADG below the 25th percentile (low performers), IQR, and above the 75th percentile (high performers). The ADG categories were used as outcomes in a multinomial logistic regression (R package nnet). Initial models included risk factors associated with P2 (TSP, breed, and birth weight category), those associated with E1 (antimicrobial exposure and health category), and factors associated with pre-weaning sampling including appetite (yes, finished milk meals between E1 andE9 or no, did not finish two or more milk meals between E1 andE9), temporal changes in log_10_ *Bifidobacterium* copies/gm at pre-weaning sampling times, and health categories at sampling times E1, E4, E9, and E14. As described previously, the goal for the final model was to include risk factors or exposure variables associated with parsimony guided by AIC and improving residual deviance. Because antimicrobial exposure at E1 was the main effect evaluated in our study, it was retained in the final model. OR with their 90% confidence intervals are reported.

#### Modeling Age to First Calving

A proportional hazards model was used to assess time to first calving. The model building approach was similar to that described for ADG assessment. Risk factors included were those associated with P2, E1, and cumulative events across the follow up period including ADG. The goal for the final model was to include risk factors or exposure variables associated with parsimony guided by a Likelihood-Ratio test. The R package survival was used to create the final model. Hazard ratios were determined with their 90% confidence intervals.

## Results

### Enrollment Data

A total of 121 heifers were enrolled in this field trial (85 Holstein and 36 Holstein-Jersey cross). No animals were removed from the study because of deteriorating health although five calves died during the pre-weaning period. The median age of enrolled calves was 8 days with an IQR of 2 which ranged between 7 and 9 days. At enrollment (E1), calves were identified by on-farm personnel as needing treatment and a similar calf not needing treatment were randomly allocated to one of the four study groups with 31, 32, 31, and 27 calves assigned to unhealthy and receiving an antimicrobial, healthy and no antimicrobial treatment, healthy and receiving an antimicrobial, and unhealthy and no antimicrobial treatment, respectively. The median value for total serum protein (TSP) for the enrolled calves was 6.2 g/dL with an IQR of 0.9 (range = 5.7–6.6g/dL). At enrollment, the median calf weight was 37 kg with an IQR of 8 kg (range = 34–42 kg).

**Figure 1:**
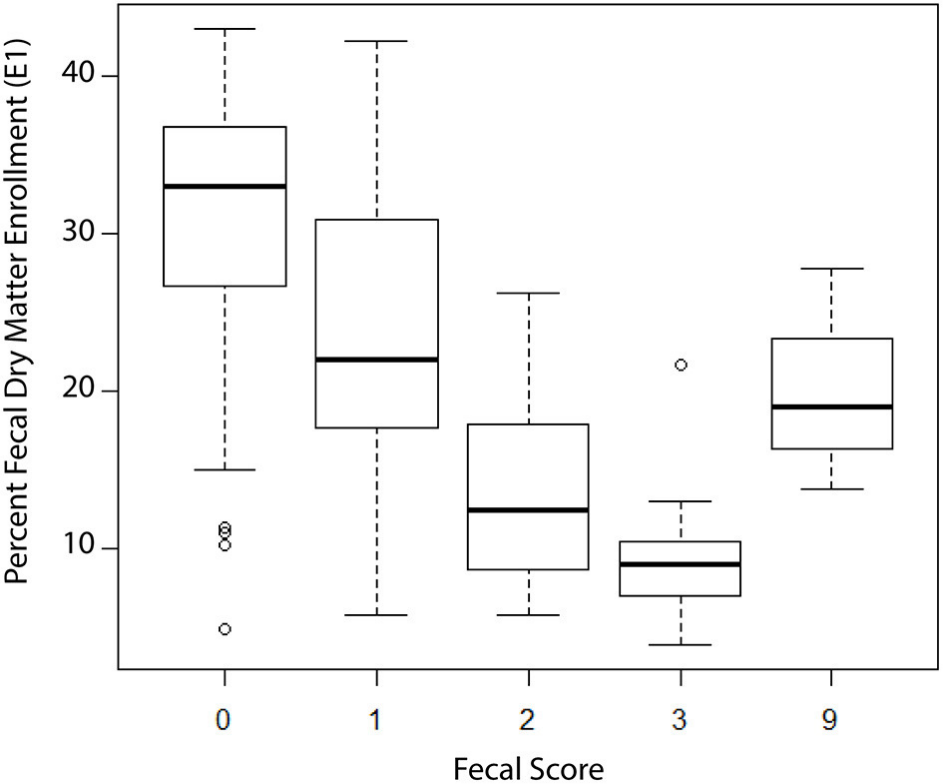
Box and whisker plots of fecal dry matter (DM) stratified by observed fecal score at enrollment of calves into the study, *n* = 121 calves. Fecal Score Definition: 0 = well-formed fecal samples; 1 = semi-formed fecal samples; 2 = loose fecal samples; 3 = watery fecal samples; 9 = not scored.

At E1 a fecal sample was collected, and a portion used to determine dry matter (DM). The fecal DM ranged from 4 to 43% with a median DM value of 26.1%. Figure 1 shows the box and whisker plots of DM by observed fecal score on day E1. Although there was variability in DM at each of the four fecal scores as well as overlap between scores, the trend was a decreasing median DM with increasing fecal scores. Based on these data, we defined a diarrhea event as a DM ≤ 17%which was near the 75th percentile for a fecal score of two and the 25th percentile for a fecal score of 1. Using DM ≤17% as a definition of diarrhea, 11/81 calves with fecal scores of 0 or 1 were reclassified as diarrhea and 6/37 calves with fecal scores of 2 or 3 were reclassified as normal.

We compared the decision that a calf was unhealthy made by on-farm personnel to objective criteria noted by study personnel at enrollment (attitude, appetite, and rectal temperature) combined with measured DM and applied the decision tool described in Table 1. At enrollment, no calves were observed with respiratory signs suggesting bovine respiratory disease (BRD). Of the 121 calves enrolled in the study, on the day of enrollment, 43 (based on DM ≤17%) were scored with diarrhea, 15 were noted as depressed, 15 did not finish the milk meal prior to enrollment, and seven calves were identified with elevated rectal temperature (≥39.4°C) by study personnel. Based on these data, 66/121 calves (54%) remained in their original enrollment groups based on farm personnel decisions (Table 2). Twenty-nine (24%) calves were reclassified as uncomplicated diarrhea; the majority of which were reclassified from the original unhealthy category. Those reclassified calves represented the greatest change in the original risk classification where only 20/58 (34%) remained objectively classified as unhealthy (sick). For all subsequent analyses, calves were allocated to one of six objective disease categories as antibiotic treated or not in the health categories sick, uncomplicated diarrhea, and healthy.

**Table 2:** Comparison of study group allocations based on farm personnel decisions and post-enrollment criteria based on symptoms for 121 pre-weaning dairy calves.

**Health category determined by farm personnel and random treatment assignment at enrollment**	**Post-enrollment health category based on symptoms (Table 1) and treatment assignment at enrollment**						
	**Sick AM^a^**	**Sick No AM**	**Uncomplicated diarrhea AM**	**Uncomplicated diarrhea No AM**	**Healthy AM**	**Healthy No AM**	**Total**
Unhealthy AM	12	0	9	0	10	0	31
Unhealthy No AM	0	8	0	13	0	6	27
Healthy AM	5	0	5	0	21	0	31
Healthy No AM	0	5	0	2	0	25	32
Total	17	13	14	15	31	31	121

a *AM = intramuscular antimicrobial administered at enrollment (3 mg/kg ampicillin trihydrate over three consecutive days)*.

Across the six treatment and health categories there was variability for median P2 calf weight and TSP determined at P2 (Table 3). Median P2 weights tended to be lower for calves at E1 classified as sick relative to healthy calves although there was considerable overlap in the range of weights within the IQR. Median TSP values across the groups ranged from 5.9 to 6.3 g/dl and would be classified as good to excellent based on recent published recommendations (25). We used the farm level TSP distribution to create categories for subsequent analyses. The TSP values equal or below the overall 1st quartile (≤5.7 g/dl) were categorized as low and values above the 1st quartile as adequate. Similarly, P2 body weight was categorized based on “light” being equal or below the 25th percentile (≤33.6 kg) and “heavy” being equal or greater than the 75th percentile (≥41.8 kg). Calves weighing within the IQR were called “medium.”

**Table 3:** Distribution of calves' total serum protein concentration (TSP), bodyweight the day after birth, and assigned TSP category (adequate >5.7 g/dL, low ≤5.7 g/dL) by *post-hoc* study group at enrollment.

**Treatment Category**	**N**	**TSP (g/dL)**		**TSP Category**		**Day 1 body weight (kg)**	
		**Median**	**IQR (range)**	**Adequate**	**Low**	**Median**	**IQR (range)**
Sick AM	17	6.3	0.5 (6.0–6.5)	13	4	35.9	6.3 (33.2–39.5)
Sick No AM	13	6.2	0.7 (5.8–6.5)	11	2	35.9	6.9 (34.5–41.4)
Uncomplicated diarrhea AM	14	6.0	0.6 (5.5–6.1)	8	6	36.8	7.8 (34.0–41.8)
Uncomplicated diarrhea No AM	15	6.0	1.0 (5.6–6.6)	10	5	36.8	5.7 (33.2–38.9)
Healthy AM	31	6.3	0.7 (5.9–6.6)	24	7	38.2	9.3 (33.4–42.7)
Healthy No AM	31	5.9	1.2 (5.5–6.7)	19	12	40.5	8.8 (34.5–42.3)
Overall	121	6.2	0.9 (5.7–6.6)	85	36	37.3	8.2 (33.6–41.8)

### *Bifidobacterium* spp. Temporal Trends and Effect of Antimicrobial Use and Illness on Those Temporal Trends

The median, IQR, and Estimated Marginal Means (EMM) for calf log_10_ *Bifidobacterium* spp. copies/gm stratified by study days are shown (Table 4). There was a temporal trend over the course of sampling with log_10_ *Bifidobacterium* spp. quantity increasing from P2 to E0 with the highest quantities at E0 and E1 and diminishing in subsequent samplings (E4, E9, and E14). This was most notable in the later samplings as *Bifidobacterium* spp. The EMM decreased ~2 logs between sampling days E1 and E14.

**Table 4:** Distribution and summary values of log_10_ *Bifidobacterium* copy number/gram fecal for 121 calves as determined by qPCR, stratified by sampling day (P2 = day 2 of age, E0 = day prior to enrollment, E1= enrollment day and 1st follow-up day, E4 = 4th follow-up day, E9 = 9th follow-up day, and E14 = 14th follow-up day.

**Sampling day**	**Median**	**IQR**	**Estimated marginal means (EMM)**	**90% CI**
P2	7.0	3.1 (5.4–8.5)	6.53	6.18–6.88
E0	8.3	2.4 (6.7–9.1)	7.77	7.41–8.12
E1	7.8	2.3 (6.6–8.9)	7.68	7.33–8.02
E4	7.5	2.6 (6.3–8.9)	7.32	6.97–7.67
E9	6.8	2.4 (5.7–8.1)	6.68	6.33–7.03
E14	5.9	1.9 (5.0–6.9)	5.50	5.15–5.85

**Figure 2:**
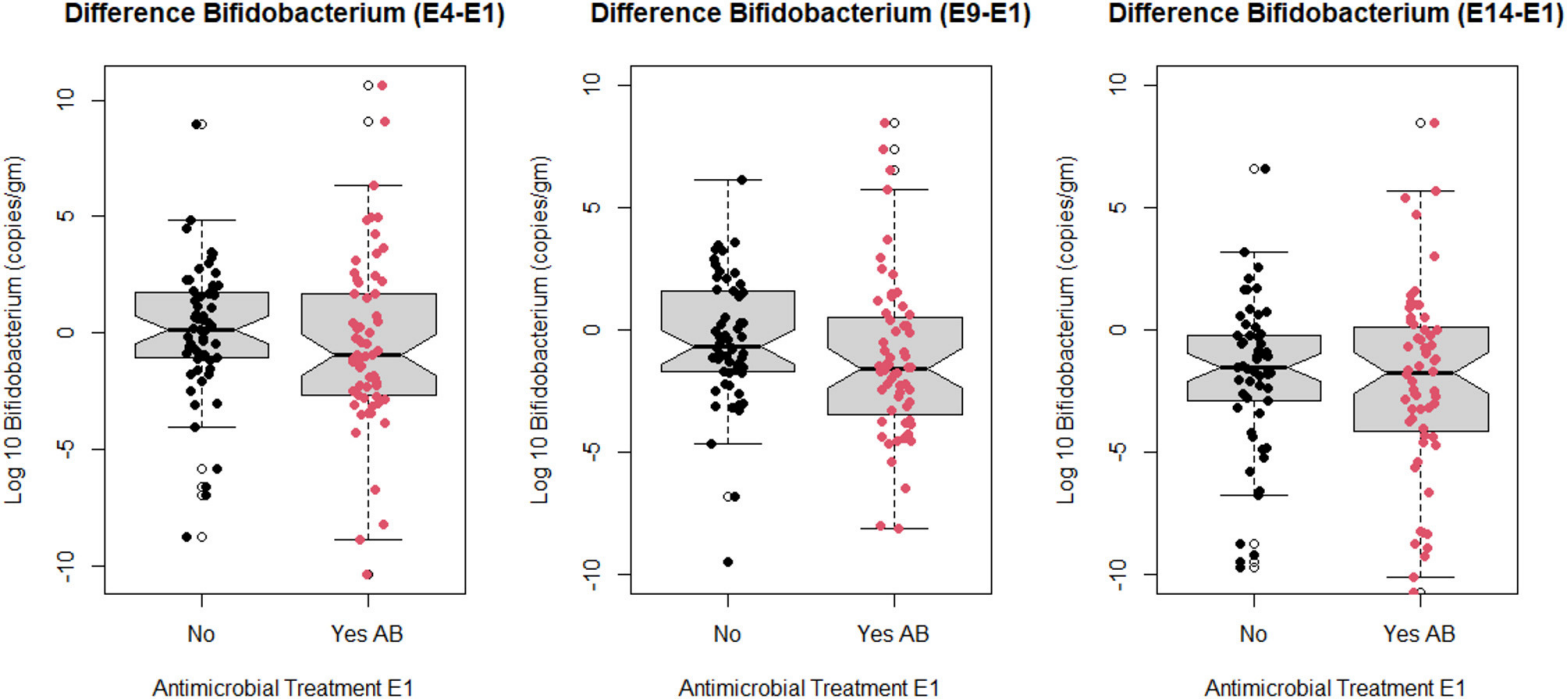
Notched box and whisker plots of change in log_10_ *Bifidobacterium* copies/gram fecal between sampling day E4, E9, E14, and sampling day E1 stratified by antimicrobial treatment group at E1 (no antimicrobial and yes antimicrobial). Points on graph represent values for study calves at each sampling point (E1 = enrollment day and 1st follow-up day, E4 = 4th follow-up day, E9 = 9th follow-up day, and E14 = 14th follow-up day).

The association of antimicrobial exposure and disease categories with temporal trend of log_10_ *Bifidobacterium* spp. (copies/gm) was assessed using the outcome measure of difference in the amount of *Bifidobacterium* spp. between sampling times (E4, E9, or E14) and E1. Figure 2 depicts notched box and whisker plots overlaid with the individual calves' difference values at each of the assessed time points. There was more visible variability in the difference values for antimicrobial treated calves relative to the untreated calves. In addition, while there was discernable overlap for IQR values between treated and untreated calves (less so at E9-E1) and overlap of notches it was clear that treated calves tended to have more relative negative values than those untreated. In addition, there was tendency for some of the distributions to be bimodal. Consequently, for subsequent data analyses at each sampling time point we created three categories as outcome variables to reflect temporal trends.

#### Difference in Log_10_ Bifidobacterium Quantity Between E4 and E1

Difference in log_10_ quantity between E4 and E1 ranged in value from −10.4 (decrease) to 10.6 (increase) with a median difference of −0.4. This difference was categorized into three outcome variables based on the quartile distribution of below the 25th percentile (<-1.98), within the IQR (−1.98–1.71), and 75th percentile and above (>1.71).

**Figure 3:**
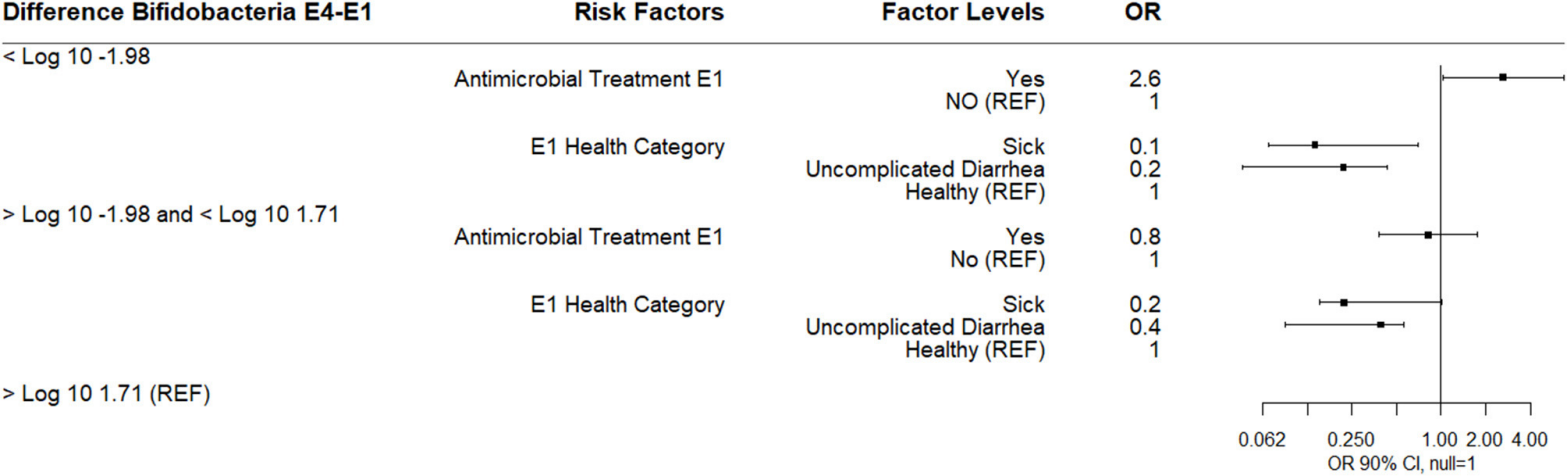
Results of multinomial logistic regression modeling the change in Log_10_ *Bifidobacterium* copies/gram fecal between sampling day E4 and sampling day E1 (enrollment). Odds ratios and 90% confidence intervals are shown. E1 antimicrobial categories were defined as: yes (received 3-day course of intramuscular ampicillin) or no (did not receive an antimicrobial). E1 health categories were defined as: sick (diarrhea with not finishing milk meal or depressed attitude), uncomplicated diarrhea (diarrhea with no additional clinical signs), and healthy.

A multinomial logistic regression using these difference categories in log_10_ *Bifidobacterium* spp. copies/gm between sampling days E4 and E1 as the outcome (reference group >1.71 log_10_ change in *Bifidobacterium* copies/gm) and treatment group at E1 (reference group = “did not receive antimicrobials”) and objective disease categories at E1 and E4 as risk factors was determined. Calves receiving an antibiotic at E1 for 3 days were more likely to have a 1.98 log_10_ or greater decrease in *Bifidobacterium* copies/gm compared to calves receiving no antimicrobial therapy at E1. Calves that were categorized as sick at E1 or uncomplicated diarrhea were less likely to be in either the lowest or IQR *Bifidobacterium* spp. difference categories relative to healthy calves suggesting that sick calves at E1 had lower baseline than healthy calves (Figure 3). Breed was not a risk factor in this model or in any of the subsequent models.

#### Difference in Log_10_ Bifidobacterium Quantity Between E9 and E1

The difference in log_10_ quantity between E9 and E1 ranged in value from −9.53 to 8.48 with a median difference of −1.11 reflecting the overall trend of decreasing *Bifidobacterium* spp. over the sampling periods. This difference was also categorized into three outcome variables based on the quartile distribution of below the 25th percentile (<-2.54), IQR (−2.54–0.74), and 75th percentile (>0.74–reference group).

**Figure 4:**
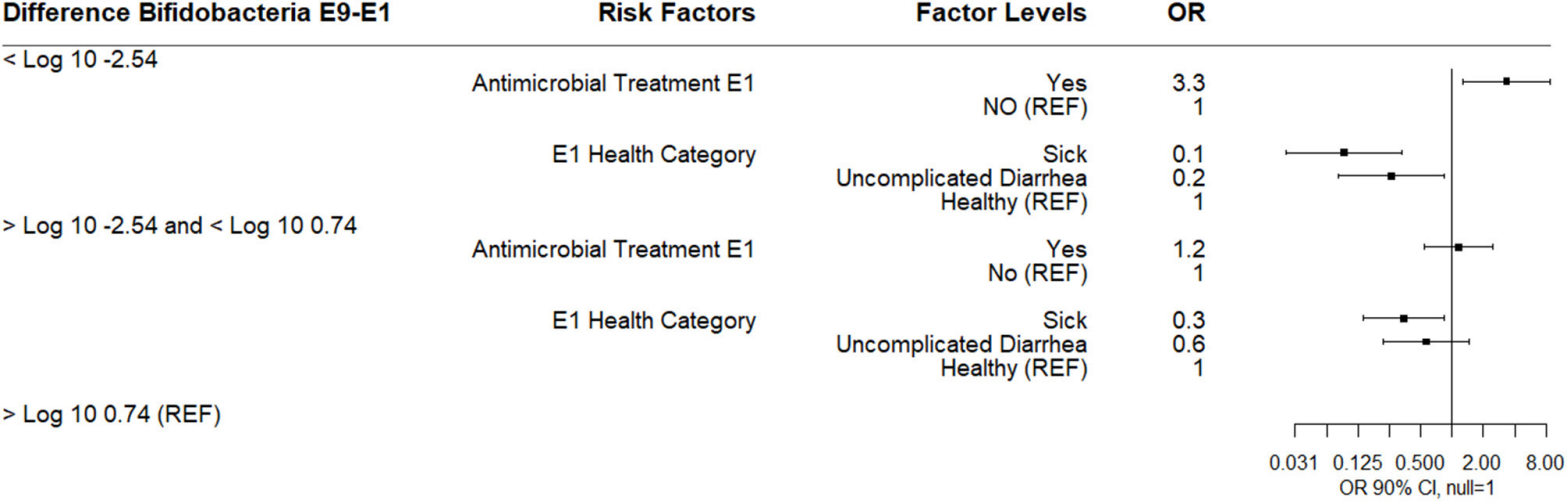
Results of multinomial logistic regression modeling the change in Log_10_ *Bifidobacterium* copies/gram fecal between sampling day E9 and sampling day E1. Odds ratios and 90% confidence intervals are shown.

A multinomial logistic regression using the E9-E1 *Bifidobacterium* difference categories and risk factors of E1 antimicrobial category and health categories at E1, E4, and E9 found that calves receiving an antibiotic at E1 for 3 days were more likely to experience a 2.5 log_10_ or greater decrease in *Bifidobacterium* spp. between E1 to E9 compared to calves not receiving an antimicrobial. Calves classified sick at E1 were associated with a decreased likelihood of either a 2.5 log_10_ or greater decrease in *Bifidobacterium* spp. between E1 to E9 or in the IQR E9-E1 *Bifidobacterium* difference category compared to healthy calves. Calves with uncomplicated diarrhea at E1 were also less likely to experience a 2.5 log_10_ or greater decrease in *Bifidobacterium* spp. (Figure 4). There was no association between disease categories at E4 or E9 on *Bifidobacterium* difference category.

#### Difference in Log_10_ Bifidobacterium Quantity Between E14 and E1

The difference in log_10_ *Bifidobacterium* spp. quantity between E14 and E1 ranged in value from −10.8 to 8.5 with a median difference of −1.63 which reflected the overall trend that E14 sampling had the lowest median value for *Bifidobacterium* spp. content. This difference was categorized into three outcome variables based on the quartile distribution of below the 25th percentile (<−3.66), IQR [−3.66–(−0.033)], and above the 75th percentile (>−0.033).

**Figure 5:**
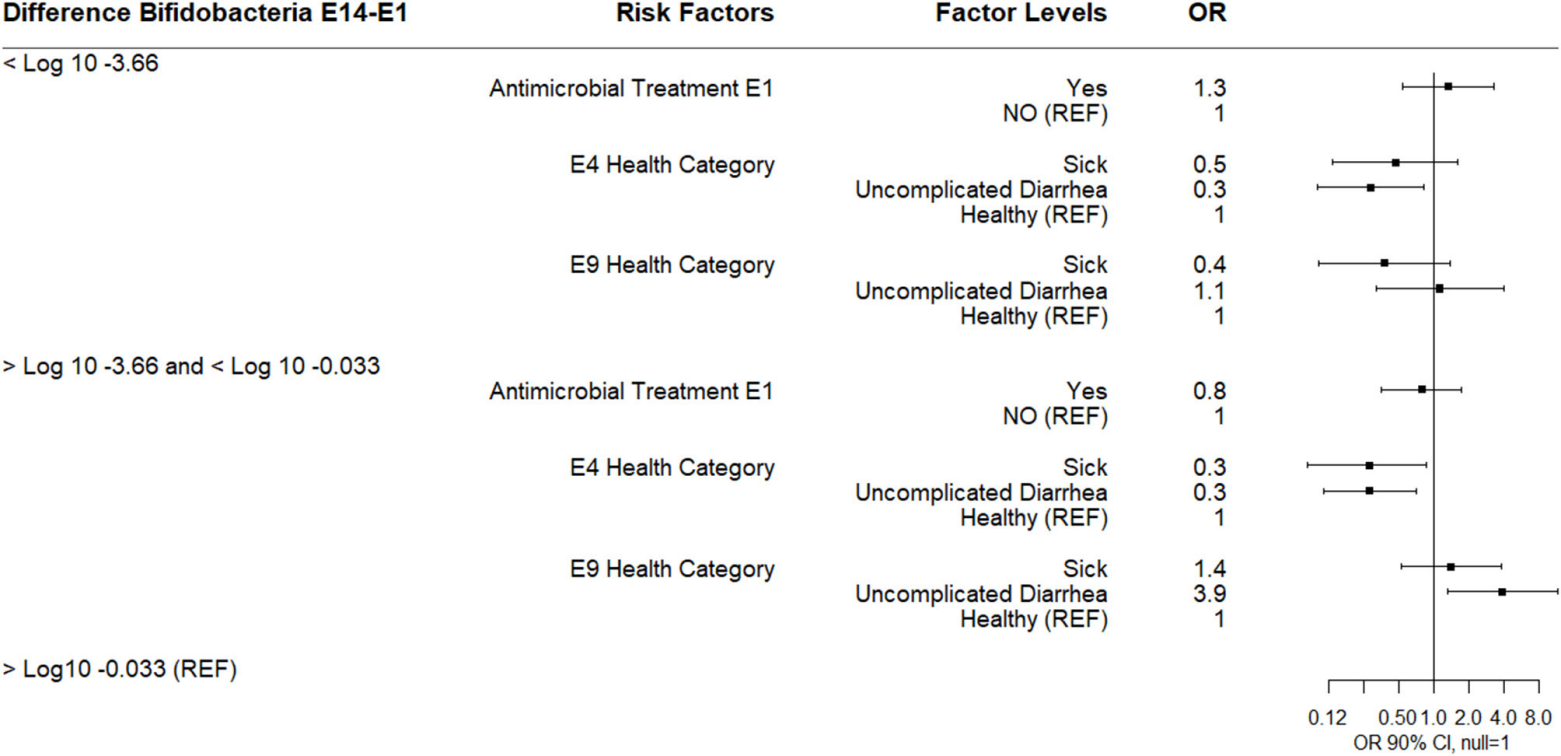
Results of multinomial logistic regression modeling the change in Log_10_ *Bifidobacterium* copies/gram fecal between sampling day E14 and sampling day E1. Odds ratios and 90% confidence intervals are shown. E4 and E9 health categories were defined as: sick (diarrhea with not finishing milk meal and/or depressed attitude), uncomplicated diarrhea (diarrhea with no additional clinical signs), and healthy.

A multinomial logistic regression using the E14-E1 *Bifidobacterium* difference categories found that calves categorized with uncomplicated diarrhea at E4 were less likely to experience a 3.6 log_10_ or greater decrease in *Bifidobacterium* spp. or in the IQR E14-E1 *Bifidobacterium* difference category compared to healthy calves. Sick calves at E4 were also less likely to be in the IQR *Bifidobacterium category*. In contrast, calves with uncomplicated diarrhea at E9 were more likely to be in the IQR *Bifidobacterium* category compared to healthy calves (Figure 5). There was no effect of antimicrobial exposure at E1 on the difference in log_10_ *Bifidobacterium* spp. between E14 and E1.

### Risk Factors Associated With Health Categories at E1, E4, E9, and E14

#### Risk Factors Associated With Health–E1

**Figure 6:**
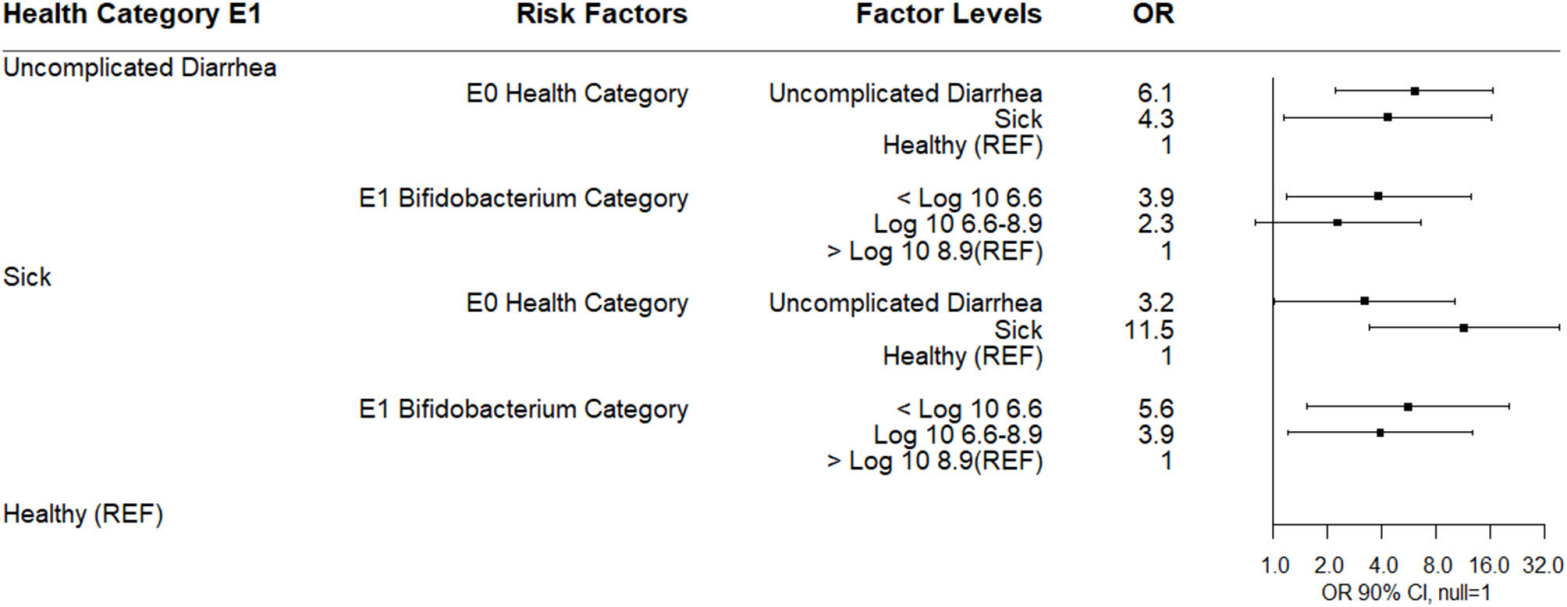
Results of multinomial logistic regression modeling risk factors for health category at sampling day E1. Odds ratios and 90% confidence intervals are shown. E0 health categories were defined as: sick (diarrhea with not finishing milk meal and/or depressed attitude), uncomplicated diarrhea (diarrhea with no additional clinical signs), and healthy.

The results of a multinomial logistic regression analysis for risks for disease category at enrollment (E1) as the outcome are shown (Figure 6). Calves classified as uncomplicated diarrhea were more likely to be in the lowest quantity of three categories for log_10_ *Bifidobacterium* (<6.6 log_10_ copies/gm) at E1 relative to healthy calves (quartile distributions shown in Table 4). Calves were also more likely to be categorized at enrollment as uncomplicated diarrhea if they were classified uncomplicated diarrhea or sick at E0. Calves categorized as sick compared to healthy on enrollment day were also associated with being in the lowest category for log_10_ fecal *Bifidobacterium* spp. at E1 or within the IQR for log_10_ fecal *Bifidobacterium* spp. on E1. If calves were classified as sick at E0 they were likely to be classified as sick at enrollment.

#### Risk Factors Associated With Health–E4

**Figure 7:**
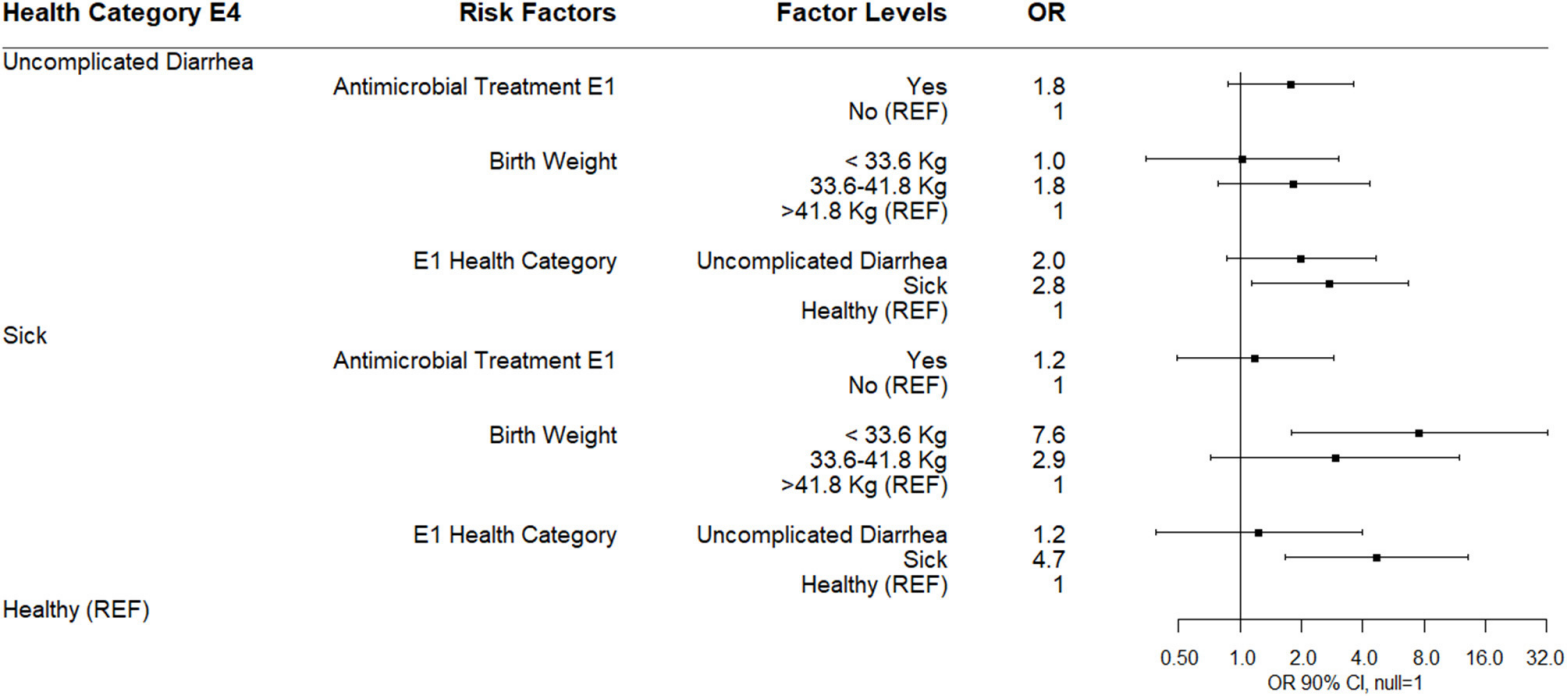
Results of multinomial logistic regression modeling health category at sampling day E4. Odds ratios and 90% confidence intervals are shown.

The results of a multinomial logistic regression for risks for health category at sampling day E4 (4 days following enrollment or 1 day after the last antimicrobial treatment) are shown (Figure 7). Calves classified as uncomplicated diarrhea compared to healthy at E4 were associated with being classified as sick at E1. Calves classified as sick compared to healthy at E4 were also associated with being classified as sick at E1 and being in the lowest birth weight classification. Neither antimicrobial treatment at E1 nor log_10_ *Bifidobacterium* copies/gm at E4 were associated with health category at E4 (Figure 6).

#### Risk Factors Associated With Health–E9 and E14

**Figure 8:**
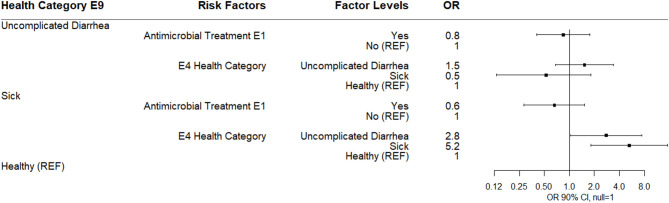
Results of multinomial logistic regression modeling health category at sampling day E9. Odds ratios and 90% confidence intervals are shown.

The multinomial logistic regression results for risks for health category at sampling day E9 (9 days post-enrollment) are shown (Figure 8). Calves categorized as sick at E9 were more likely to be observed as uncomplicated diarrhea or sick at E4 compared to healthy calves. No risk factors for uncomplicated diarrhea were noted. No association between disease category at E9 and antibiotic use at E1 or log_10_ E9 *Bifidobacterium* was observed.

**Figure 9:**
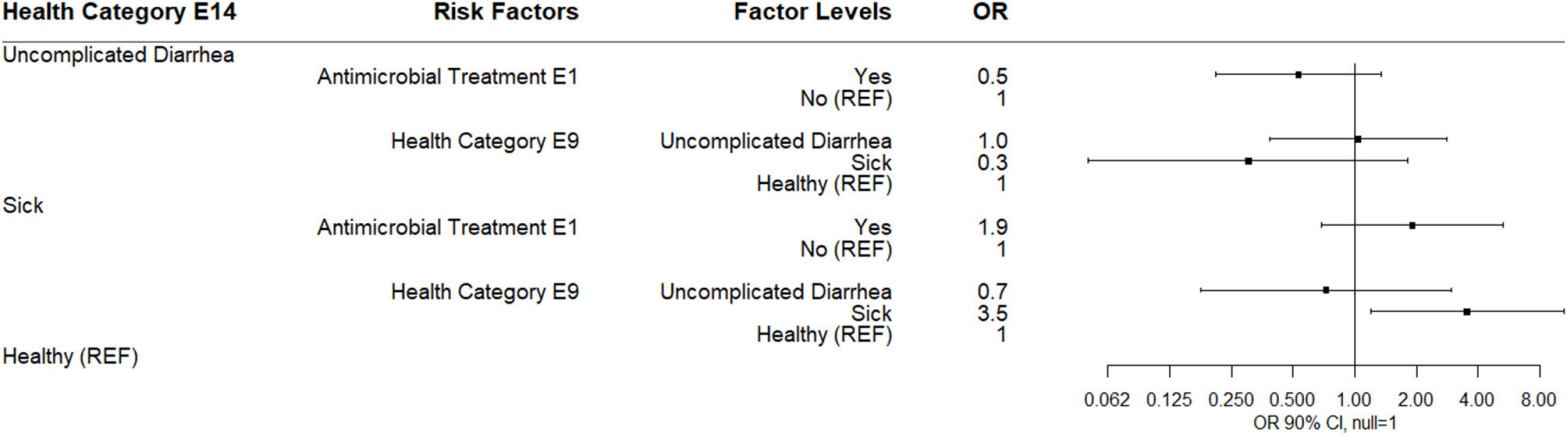
Results of multinomial logistic regression modeling health category at sampling day E14. Odds ratios and 90% confidence intervals are shown.

By E14 (14 days post-enrollment), 74% of study calves were categorized as healthy. The only observed association in a multinomial logistic regression was for calves categorized as sick at E14 were more likely to have been categorized sick at E9. Neither antimicrobial treatment at E1 nor log_10_ *Bifidobacterium* at E14 were associated with health category at E14 (Figure 9).

### Impact on Pre-weaning Average Daily Weight Gain

Study calves were weighed at arrival to the calf rearing area (day 1 after birth) and again at weaning. The average pre-weaning period for calves was 61 days (median = 60 days). The average weaning weight was 80.7 kg (median = 80.9 kg) and ADG was 0.7 kg (median = 0.7 kg). For subsequent analysis, ADG was categorized into three levels based on quartile distribution: low <0.6 kg/day (25th percentile), medium ≥0.6 kg/day and <0.8 kg/day (IQR), and high ≥0.8 kg/day (75th percentile).

**Figure 10:**
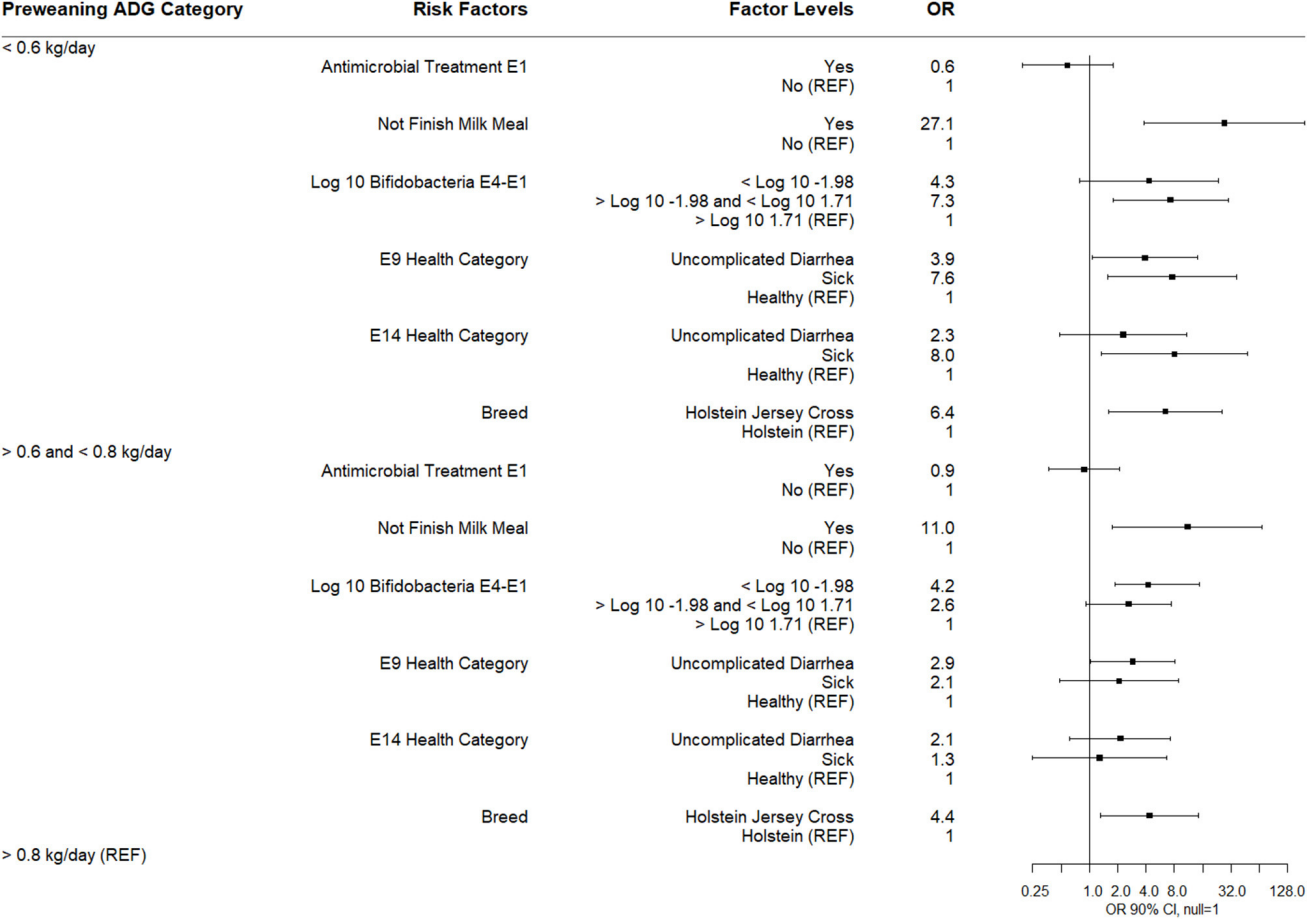
Results of multinomial logistic regression modeling pre-weaning average daily gain. Odds ratios and 90% confidence intervals are shown. Not finish milk meal was defined as: yes (finished milk meals between E1 and E9) and no (did not finish 2 or more milk meals between E1 and E9).

The results of a multinomial logistic regression for risks for ADG category as the dependent variable (high = reference group) are shown (Figure 10). Calves not finishing their milk meal more than two times between sampling times E1-E9, calves categorized as sick or with uncomplicated diarrhea at E9 or sick at E14, and calves categorized in IQR category for the difference in *Bifidobacterium* spp. quantity between E4 and E1, and Holstein Jersey cross were associated with being in the low pre-weaning ADG category. Calves not finishing their milk meal more than two times, calves categorized below the 25th percentile for difference in *Bifidobacterium* spp. quantity between E4 and E1, calves categorized with uncomplicated diarrhea at E9, and Holstein Jersey cross were associated with the medium ADG category. There was no association of E1 antimicrobial treatment with ADG category.

### Post-Weaning Events

Using on-farm records, calves were followed post-weaning to assess effect of treatment and pre-weaning events on survival in the herd and time to first calving. Of the 121 calves originally enrolled in the study, 97 entered their first lactation, 14 had died, seven were sold, and two were lost to follow-up post-weaning. Of the 14 that died, five died during the pre-weaning period, two died within 7 days following weaning, five died between 100 and 170 days of age, and one died at 558 days of age. For the 97 study animals that calved, the median and mean age at first calving was 22.5 months with the IQR being ~30 days. None of the pre-weaning variables (disease status at sampling points, *Bifidobacterium* quantity, E1 antimicrobial treatment, ADG category, breed, or TSP category) were associated with age at first calving.

## Discussion

To our knowledge this is the first study of the effects of parenteral antimicrobials given to healthy as well as unhealthy pre-weaning calves on fecal *Bifidobacterium* quantity and health outcomes. On farm detection of calf disease is challenging and in this study was inconsistent in its application. This inconsistency makes it difficult to use farm records to make management decisions on efficacy of treatments. Antimicrobials impacted the temporal pattern of *Bifidobacterium* succession in both healthy and sick calves through 9 days following a 3-day course of parenteral antimicrobials but had no impact on health outcomes or growth after treatment. The temporal pattern of *Bifidobacterium* and health assessments made during the study were closely aligned with calves classified as sick being associated with lower quantities of fecal *Bifidobacterium* and previously being identified as sick.

Identification of the frequent misclassification of illness was an important finding. Based on comparisons with clinical observations, we could not rely on farm personnel decisions for what defined a sick calf. This finding has been observed elsewhere (5) and cautions on farm researchers and dairy advisors to question the utility of farm treatment records to identify or evaluate farm morbidity. The on-farm criteria for determining a sick animal should be as objective as possible and clearly defined to be consistently applied by on-farm personnel charged with health assessments. Even clinical scoring systems have potential for misclassification when compared to more objective measures, such as dry matter content of feces vs. fecal score. The trend we saw with fecal score and fecal DM has been observed by others who used DM to normalize estimates of parasite load (26) and points out the underlying variability of DM associated with a fecal score. In addition, our findings indicate that levels of illness severity should be considered both in classification of a disease as well as to identify treatment options. Diarrhea is a symptom and not a disease and there appear to be gradations of severity that are not obvious through observation. Just as with dairy cow mastitis severity scoring, the outcomes and appropriate therapies for levels of diarrhea severity may differ (27) and this points to the importance of developing quick and easy point of care diagnostics to augment observation and intuition.

Follow-up sampling of neonatal calves revealed a temporal trend over the course of sampling with log_10_ *Bifidobacterium* quantity increasing from day 2 of life to the day of enrollment (about 8 days of age) with the highest quantities at the day before and the day of enrollment and diminishing in subsequent samplings (4, 9, and 14 days post-enrollment). Others have reported a rise in *Bifidobacterium* spp. fecal bacteria count from the first to the third week of life and a decline in week 4 and 5 (28) and that the relative abundance of *Bifidobacterium* appears to decrease with the age of the calf from day 7–14 (29).

The introduction of new feed is likely to have an influence on the bacterial species presented to the GIT. Despite the fact that the calves at the enrollment age are consuming mostly milk or milk replacer, calves' consumption of starter feed doubles in the first 2 weeks of life and by 3 weeks of age, triples in quantity compared to consumption in the first week of life (30). These diet changes affect both the lower GIT as well as establishment of the rumen bacterial community (31). In addition, fecal *Bifidobacterium* dynamics appears dependent on diet, with higher counts found in all milk diets compared to diets with milk and grains (32).

Regardless of health status, calves in our study receiving a 3-day course of parenteral antimicrobial experienced a large decrease in *Bifidobacterium* compared to untreated healthy calves which demonstrated an increase in *Bifidobacterium* between E1 and E4. The differences associated with antimicrobial use might indicate a destabilization of the gut microbiota. A human neonatal study evaluating the impact of parenteral antimicrobials (ampicillin/gentamicin) on fecal *Bifidobacterium* showed a similar effect to those in our study (33). Using a different study design, Ma and others (7) investigated disturbances to the gut microbiome and reported that the use of antibiotics early in a calf's life delayed the development of microbial diversity. They noted that a gut microbiome with greater stability was more resistant to outside disturbances. In another study, when oxytetracycline was fed at different levels compared to controls, the calf microbiota composition was more affected by time and not antibiotic level (15).

One of our study objectives was to describe the impact of antimicrobial therapy on health outcomes. Across all the pre-weaning follow-up sampling periods (E4, E9, E14), antimicrobial therapy was not associated with post-treatment calf health. Calves classified as sick at enrollment (E1) were more likely to be classified as sick at E4 regardless of E1 treatment group. This suggests that antimicrobial treatment had little or no impact on the course of disease. There was also no evidence that antimicrobial treatment affected health outcomes for those healthy calves that were selected to receive antimicrobials. This trend held true for all the follow-up timepoints as calves classified as sick at one timepoint were associated with being sick at the previous timepoint.

In our study, enrollment health category was associated with *Bifidobacterium* quantity; calves with either uncomplicated diarrhea or classified as sick were associated with the lowest quartile of E1 log_10_ fecal *Bifidobacterium* relative to healthy calves. Others have reported a similar finding, i.e., higher levels of *Bifidobacterium* spp. were associated with healthy vs. diarrheic calves. Although in the same study a second farm had other bacterial species associated with health (34), suggesting that microbiota is farm specific. In another study, having diarrhea was associated with a fluctuation in microbial diversity and temporal stability of the fecal microbiota was considered best in healthy calves compared to sick (7). In our study, we were not able to assign cause and effect, i.e., whether abnormal health status was a consequence of lower quantities of *Bifidobacterium* present in feces or whether abnormal health resulted in lower quantities. It is possible that lower fecal DM associated with our classification of health reflected a decreased amount of detected *Bifidobacterium* per gram of feces for calves with low fecal dry matters though the temporal trends we observed in our study were similar to those reported elsewhere (29).

Antimicrobial treatment did not affect either ADG or post-weaning events associated with mortality or days to first calving. There were associations of not finishing milk meals and being classified as sick at E9 and E14 on decreased ADG. There was no consistent finding associated with *Bifidobacterium* change between E4 and E1, though a depressed change was associated with lower ADG. We did not monitor and collect daily health scores on calves between E14 and weaning and could not account for their possible impact on ADG, but it is important to note that recorded sick events at E9 and E14 as well as not completing milk meals between E1 and E9 had impacts on ADG, i.e., sick calves did not appear to catch up to their healthy peers following the early negative pre-weaning events.

In summary, health and treatment decision making on the farm is often subjective particularly when determining whether antibiotic treatment is appropriate for an animal. These data illustrate a misalignment between clinical observations made by investigators and the initiation of antibiotic treatment by farm personnel. This presents an opportunity for calf treaters and veterinarians to develop and evaluate protocols for disease detection. Antimicrobials used in our study accelerated the temporal trend of decreasing *Bifidobacterium* and by themselves had no impact on the course of disease. Although it is unclear if these observations are related, it does suggest that discretionary use of antimicrobial therapy should be guided by veterinary input and monitoring and highlights the need for point of care diagnostics to better define gradients of disease.

## Data Availability Statement

The datasets presented in this study can be found in online repositories. The names of the repository/repositories and accession number(s) can be found at: https://doi.org/10.7273/k0hn-gy79.

## Ethics Statement

The research protocol was reviewed and approved by the Institutional Animal Care and use Committee of Washington State University (ASAF 04925). All protocols involving calves housed on the commercial dairy farm were authorized by the farm owner, who was aware of all procedures. Written informed consent was obtained from the owners for the participation of their animals in this study.

## Author Contributions

OO'K participated in project design and implementation and contributed to all versions of the manuscript. DM participated in project implementation and contributed to all versions of the manuscript. CM participated in project implementation and contributed to the final version of the manuscript. WS participated in project design and implementation and contributed to all versions of the manuscript. All authors have read and approved of the submitted version of the manuscript.

## Conflict of Interest

The authors declare that this study received funding from Boehringer Ingelheim Vetmedica, Inc. The funder was not involved in the study design, collection, analysis, interpretation of data, the writing of this article or the decision to submit it for publication.
